# The genome sequence of *Jasione montana* L., 1753 (Asterales: Campanulaceae)

**DOI:** 10.12688/wellcomeopenres.26778.1

**Published:** 2026-06-13

**Authors:** Maarten J. M. Christenhusz, Ilia J. Leitch, Michael F. Fay, Royal Botanic Gardens Kew Genome Acquisition Lab, Plant Genome Sizing Collective, Darwin Tree of Life Barcoding collective, Wellcome Sanger Institute Tree of Life Management, Samples and Laboratory team, Wellcome Sanger Institute Scientific Operations: Sequencing Operations, Wellcome Sanger Institute Tree of Life Core Informatics team, Tree of Life Core Informatics collective, Darwin Tree of Life Consortium

**Affiliations:** 1 Royal Botanic Gardens Kew, Richmond, England, UK; 2 Botanical Society of Britain and Ireland, St Albans, Hertfordshire, England, UK

**Keywords:** Jasione montana, sheep’s-bit, genome sequence, chromosomal, Asterales

## Abstract

We present a genome assembly of *Jasione montana* (sheep’s-bit; Streptophyta; Magnoliopsida; Asterales; Campanulaceae). The assembly consists of two haplotypes with total lengths of 1 282.03 megabases and 1 294.56 megabases. Most of haplotype 1 (98.9%) is scaffolded into 6 chromosomal pseudomolecules. Haplotype 2 was assembled to scaffold level. The mitochondrial sequence has a length of 321.12 kilobases and the plastid genome assembly has a length of 200.38 kilobases. This assembly was generated as part of the Darwin Tree of Life project, which produces reference genomes for eukaryotic species found in Britain and Ireland.

## Species taxonomy

Eukaryota; Viridiplantae; Streptophyta; Streptophytina; Embryophyta; Tracheophyta; Euphyllophyta; Spermatophyta; Magnoliopsida; Mesangiospermae; eudicotyledons; Gunneridae; Pentapetalae; asterids; campanulids; Asterales; Campanulaceae; *Jasione*; *Jasione montana* L., 1753 (NCBI:txid239452).

## Background

As part of the Darwin Tree of Life Project, a collaborative effort to sequence all named eukaryotic species in the Atlantic Archipelago of Britain and Ireland, we sequenced the genome of sheep’s-bit, *Jasione montana* L. Here we present a chromosomally complete genome sequence based on a wild specimen from Trefor, Gwynedd, Wales, UK.

*Jasione montana* is an annual or biennial herb although in Britain and Ireland, it is normally a partially winter-green biennial. The plant itself is pubescent with oblong to oblanceolate leaves and its inflorescence comprises a congested terminal head of tubular, actinomorphic flowers with a blue corolla ( Stace, 2010). When bruised, all plant parts have a strong, unpleasant smell ( Grieve, 1994).

The native range of *J. montana* is Europe and north-west Africa. It also occurs as an introduction in the Azores and the eastern USA. It includes three subspecies: one (subsp. *montana*) is widespread, whereas subsp. *cornuta* (Ball) Greuter & Burdet is endemic to Morocco, and subsp. *paivae* Horjales & Rubido is endemic to Spain ( POWO, 2026). In Britain, the stronghold of subsp. *montana* is now in coastal western Britain, having disappeared from many central and eastern sites during the 20th century. In Ireland, it is predominantly coastal ( BSBI, 2026).

In coastal sites, it is a plant of sea cliffs, maritime grasslands and stabilised sand dunes on acidic, shallow, well-drained soils, whereas inland sites include dry heathland, stone walls, hedge banks and railway cuttings. Open sites and recently burnt ground are also frequently colonised. Overall, it is predominantly a lowland species, occurring from sea level to nearly 1 000 m in Co. Kerry, Ireland ( BSBI, 2026).

The flower heads superficially resemble those found in species of scabious ( *Scabiosa* L. and *Succisa* Haller; Caprifoliaceae), and an alternative common name for *J. montana* is sheep’s-bit scabious (e.g. North Wales Wildlife Trust, 2026). However, the flowers can be distinguished by their fused stamens (in contrast to free in *Scabiosa* and *Succisa*).

The sporophytic chromosome count has been reported as 2 *n* = 12 (e.g. Henniges *et al.*, 2022). Most of the assembly presented here is scaffolded into 6 chromosomal pseudomolecules, consistent with the reported chromosome count. This study provides a genomic resource for comparative phylogenomics and classification in Campanulaceae.

## Methods

### Sample acquisition, flow cytometry and DNA barcoding

**Figure 1.**
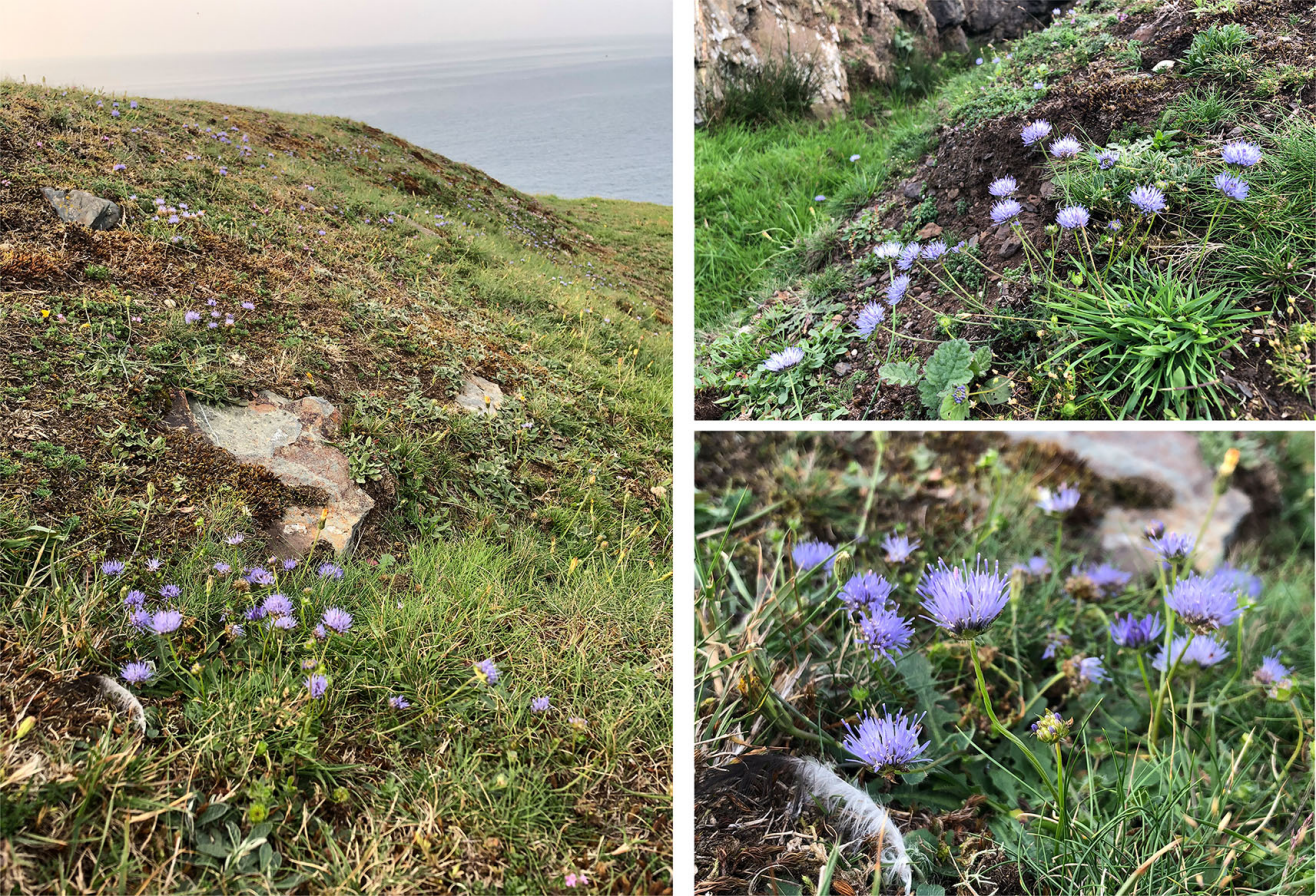
Photographs showing the habitat and habit of the *Jasione montana* (daJasMont1) specimen from which samples were taken for genome sequencing.

A specimen of *Jasione montana* (specimen ID KDTOL10350, ToLID daJasMont1; Figure 1) was used for genome sequencing. It was collected from Trefor, Gwynedd, Wales, UK (latitude 52.9938, longitude −4.4403) on 2021-08-12. The specimen was collected and identified by Maarten Christenhusz (Herbarium voucher: M. Christenhusz 9269 (K)).

The genome size was estimated by flow cytometry following the ‘one-step’ method outlined in Pellicer *et al*. (2021) and using propidium iodide as the fluorochrome. The General Purpose Buffer (GPB) supplemented with 3% PVP was used for isolation of nuclei ( Loureiro *et al.*, 2007), and the internal calibration standard was *Petroselinum crispum* ‘Champion Moss Curled’ with an assumed 1C-value of 2 200 Mb ( Obermayer *et al.*, 2002).

The initial identification was verified by an additional DNA barcoding process according to the framework developed by Twyford *et al*. (2024). Part of the plant specimen was preserved in silica gel desiccant ( Chase & Hills, 1991). DNA extracted from the dried plant was amplified by PCR for standard barcode markers, with the amplicons sequenced and compared to public sequence databases including GenBank and the Barcode of Life Database (BOLD) ( Ratnasingham & Hebert, 2007). Following whole genome sequence generation, the relevant DNA barcode region was also used alongside the initial barcoding data for sample tracking at the WSI ( Twyford *et al.*, 2024). The standard operating procedures for Darwin Tree of Life barcoding are available on protocols.io.

### Nucleic acid extraction

Protocols for high molecular weight (HMW) DNA extraction developed at the Wellcome Sanger Institute (WSI) Tree of Life Core Laboratory are available on protocols.io ( Howard *et al.*, 2025). The daJasMont1 sample was weighed and triaged to determine the appropriate extraction protocol. For HMW DNA extraction, 52 mg of flower tissue was used. Tissue from the flower was homogenised by cryogenic bead beating. Sheared DNA was purified by automated SPRI (solid-phase reversible immobilisation), using AMPure PB beads (Pacific Biosciences) and the Thermo Fisher KingFisher™ Apex to eliminate shorter fragments and concentrate the DNA. The concentration of the sheared and purified DNA was assessed using a Nanodrop spectrophotometer and Qubit Fluorometer using the Qubit dsDNA High Sensitivity Assay kit. Fragment size distribution was evaluated by running the sample on the FemtoPulse system. For this sample, the final post-shearing DNA had a Qubit concentration of 20.8 ng/μL and a yield of 2 808.00 ng. The 260/280 spectrophotometric ratio was 1.79, and the 260/230 ratio was 1.48. The Genomic Quality Number (GQN) was 5.4.

### PacBio HiFi library preparation and sequencing

Library preparation and sequencing were performed at the WSI Scientific Operations core. Libraries were prepared using the SMRTbell Prep Kit 3.0 (Pacific Biosciences) according to the manufacturer’s instructions. The kit includes reagents for end repair/A-tailing, adapter ligation, post-ligation SMRTbell bead clean-up, and nuclease treatment. Size selection and clean-up were performed using diluted AMPure PB beads (Pacific Biosciences). DNA concentration was quantified using a Qubit Fluorometer v4.0 (ThermoFisher Scientific) and the Qubit 1X dsDNA HS assay kit. Final library fragment size was assessed with the Agilent Femto Pulse Automated Pulsed Field CE Instrument (Agilent Technologies) using the gDNA 55 kb BAC analysis kit.

The sample was sequenced on a Revio instrument (Pacific Biosciences). The prepared library was normalised to 2 nM, and 15 μL was used for making complexes. Primers were annealed and polymerases bound to generate circularised complexes, following the manufacturer’s instructions. Complexes were purified using 1.2X SMRTbell beads, then diluted to the Revio loading concentration (200–300 pM) and spiked with a Revio sequencing internal control. The sample was sequenced on a Revio 25 M SMRT cell. The SMRT Link software (Pacific Biosciences), a web-based workflow manager, was used to configure and monitor the run and to carry out primary and secondary data analysis.

### Hi-C

***Sample preparation and crosslinking***

Hi-C data were generated from the leaf tissue of daJasMont1 using the Arima-HiC v2 kit (Arima Genomics). Tissue was finely ground using the Covaris cryoPREP Dry Pulverizer (Covaris), and then subjected to nuclei isolation. Nuclei were isolated using a modified protocol based on the Qiagen QProteome Cell Compartment Kit (Qiagen), in which only the Lysis and CE2 buffers were used, with QIAshredder spin columns. After isolation, nuclei were fixed using formaldehyde to a final concentration of 2% to crosslink the DNA. The crosslinked DNA was then digested and biotinylated according to the manufacturer’s instructions. A clean-up step was performed with SPRIselect beads before library preparation. DNA concentration was quantified using the Qubit Fluorometer v4.0 (Thermo Fisher Scientific) and the Qubit HS Assay Kit, following the manufacturer’s instructions.

***Hi-C library preparation and sequencing***

Biotinylated DNA constructs were fragmented using a Covaris E220 sonicator and size selected to 400–600 bp using SPRISelect beads. DNA was enriched with Arima-HiC v2 kit Enrichment beads. End repair, A-tailing, and adapter ligation were carried out with the NEBNext Ultra II DNA Library Prep Kit (New England Biolabs), following a modified protocol where library preparation occurs while DNA remains bound to the Enrichment beads. Library amplification was performed using KAPA HiFi HotStart mix and a custom Unique Dual Index (UDI) barcode set (Integrated DNA Technologies). Depending on sample concentration and biotinylation percentage determined at the crosslinking stage, libraries were amplified with 10–16 PCR cycles. Post-PCR clean-up was performed with SPRISelect beads. Libraries were quantified using the AccuClear Ultra High Sensitivity dsDNA Standards Assay Kit (Biotium) and a FLUOstar Omega plate reader (BMG Labtech).

Prior to sequencing, libraries were normalised to 10 ng/μL. Normalised libraries were quantified again to create equimolar and/or weighted 2.8 nM pools. Pool concentrations were checked using the Agilent 4200 TapeStation (Agilent) with High Sensitivity D500 reagents before sequencing. Sequencing was performed using paired-end 150 bp reads on the Illumina NovaSeq 6000.

### Genome assembly

Prior to assembly of the PacBio HiFi reads, a database of *k*-mer counts ( *k* = 31) was generated from the filtered reads using FastK. GenomeScope2 ( Ranallo-Benavidez *et al.*, 2020) was used to analyse the *k*-mer frequency distributions, providing estimates of genome size, heterozygosity, and repeat content.

The HiFi reads were assembled using Hifiasm in Hi-C phasing mode ( Cheng *et al.*, 2021, 2022), producing two haplotypes. Hi-C reads ( Rao *et al.*, 2014) were mapped to the primary contigs using bwa-mem2 ( Vasimuddin *et al.*, 2019). Contigs were further scaffolded with Hi-C data in YaHS ( Zhou *et al.*, 2023), using the --break option for handling potential misassemblies. The scaffolded assemblies were evaluated using Gfastats ( Formenti *et al.*, 2022), BUSCO ( Manni *et al.*, 2021) and MerquryFK ( Rhie *et al.*, 2020). The organelle genomes were assembled using OATK ( Zhou *et al.*, 2025).

### Assembly curation

The assembly was decontaminated using the Assembly Screen for Cobionts and Contaminants ( ASCC) pipeline. TreeVal was used to generate the flat files and maps for use in curation. Manual curation was conducted primarily in PretextView and HiGlass ( Kerpedjiev *et al.*, 2018). Scaffolds were visually inspected and corrected as described by Howe *et al*. (2021). Manual corrections included 20 breaks, 31 joins, and removal of 21 haplotypic duplications. This reduced the scaffold count by 6.4%, reduced the scaffold N50 by 47.7%, and reduced the total assembly length by 0.6%. The curation process is described at https://gitlab.com/wtsi-grit/rapid-curation . PretextSnapshot was used to generate a Hi-C contact map of the final assembly.

### Assembly quality assessment

The MerquryFK tool ( Rhie *et al.*, 2020) was run in a Singularity container ( Kurtzer *et al.*, 2017) to evaluate *k*-mer completeness and assembly quality for both haplotypes using the *k*-mer database ( *k* = 31) computed prior to genome assembly. The analysis outputs included assembly QV scores and completeness statistics.

The genome was analysed using the BlobToolKit pipeline, a Nextflow implementation of the earlier Snakemake version ( Challis *et al.*, 2020). The pipeline aligns PacBio reads using minimap2 ( Li, 2018) and SAMtools ( Danecek *et al.*, 2021) to generate coverage tracks. It runs BUSCO ( Manni *et al.*, 2021) using lineages identified by querying NCBI datasets ( O’Leary *et al.*, 2024). For the three domain-level lineages, BUSCO genes are aligned to the UniProt Reference Proteomes database ( Bateman *et al.*, 2023) using DIAMOND blastp ( Buchfink *et al.*, 2021). The genome is divided into chunks based on the density of BUSCO genes from the closest taxonomic lineage, and each chunk is aligned to the UniProt Reference Proteomes database with DIAMOND blastx. Sequences without hits are chunked using seqtk and aligned to the NT database with blastn ( Altschul *et al.*, 1990). The BlobToolKit suite consolidates all outputs into a blobdir for visualisation. The BlobToolKit pipeline was developed using nf-core tooling ( Ewels *et al.*, 2020) and MultiQC ( Ewels *et al.*, 2016), with containerisation through Docker ( Merkel, 2014) and Singularity ( Kurtzer *et al.*, 2017).

## Genome sequence report

### Sequence data

**Figure 2.**
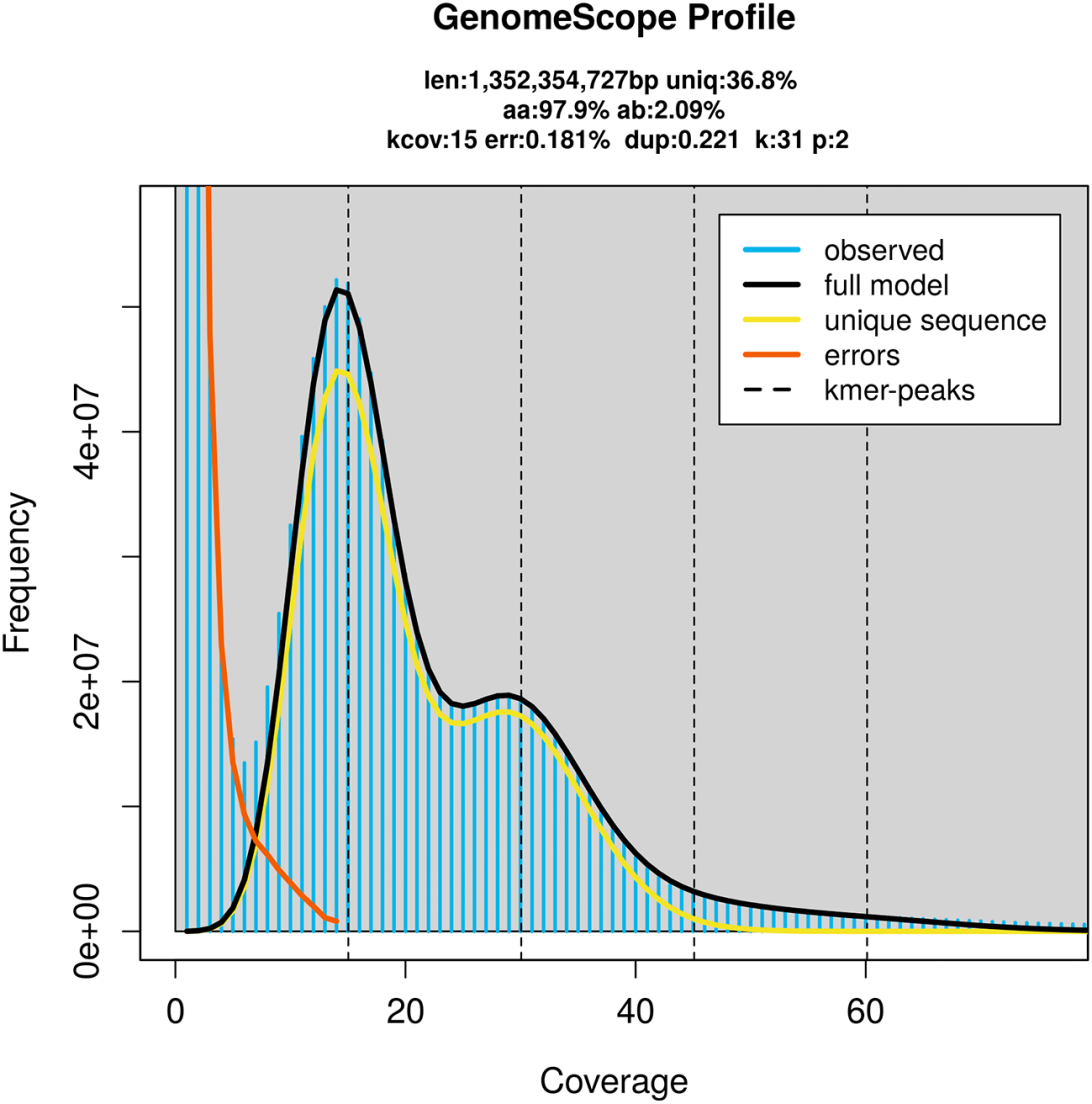
Frequency distribution of *k*-mers generated using GenomeScope2. The plot shows observed and modelled *k*-mer spectra, providing estimates of genome size, heterozygosity, and repeat content based on unassembled sequencing reads.

The genome of a specimen of *Jasione montana* was sequenced using Pacific Biosciences single-molecule HiFi long reads, generating 43.11 Gb (gigabases) from 3.57 million reads, which were used to assemble the genome. GenomeScope2.0 analysis estimated the haploid genome size at 1 359.34 Mb, with a heterozygosity of 2.09% and repeat content of 63.35% ( Figure 2). Using flow cytometry, the genome size (1C-value) of the sample was estimated to be 1.81 pg, equivalent to 1 770.00 Mb. These estimates guided expectations for the assembly. Based on the estimated genome size, the sequencing data provided approximately 30× coverage. Hi-C sequencing produced 330.69 Gb from 1 095.01 million reads, which were used to scaffold the assembly. Table 1 summarises the specimen and sequencing details.

**Table 1.** Specimen and sequencing data for *Jasione montana* (BioProject PRJEB74169).

Platform	PacBio HiFi	Hi-C
**ToLID**	daJasMont1	daJasMont1
**Specimen ID**	KDTOL10350	KDTOL10350
**BioSample (source individual)**	SAMEA10369646	SAMEA10369646
**BioSample (tissue)**	SAMEA10369695	SAMEA10369694
**Tissue**	flower	leaf
**Instrument**	Revio	Illumina NovaSeq 6000
**Run accessions**	ERR12779284; ERR12779283	ERR12791518
**Read count total**	3.57 million reads	1 095.01 million read pairs
**Base count total**	43.11 Gb	330.69 Gb

### Assembly statistics

The genome was assembled into two haplotypes using Hi-C phasing. Haplotype 1 was curated to chromosome level, while haplotype 2 was assembled to scaffold level. The final assembly has a total length of 1 282.03 Mb in 448 scaffolds, with 253 gaps, and a scaffold N50 of 211.23 Mb ( Table 2).

**Table 2.** Genome assembly statistics for *Jasione montana.*

Genome assembly	Haplotype 1	Haplotype 2
**Assembly name**	daJasMont1.hap1.1	daJasMont1.hap2.1
**Assembly accession**	GCA_964059625.1	GCA_964106945.1
**Assembly level**	chromosome	scaffold
**Span (Mb)**	1 282.03	1 294.56
**Number of chromosomes**	6	-
**Number of contigs**	701	485
**Contig N50**	9.32 Mb	9.85 Mb
**Number of scaffolds**	448	292
**Scaffold N50**	211.23 Mb	393.42 Mb
**Longest scaffold length (Mb)**	239.47	441.38
**Organelles**	Mitochondrial genome: 321.12 kb; Plastid genome: 200.38 kb	-

**Figure 3.**
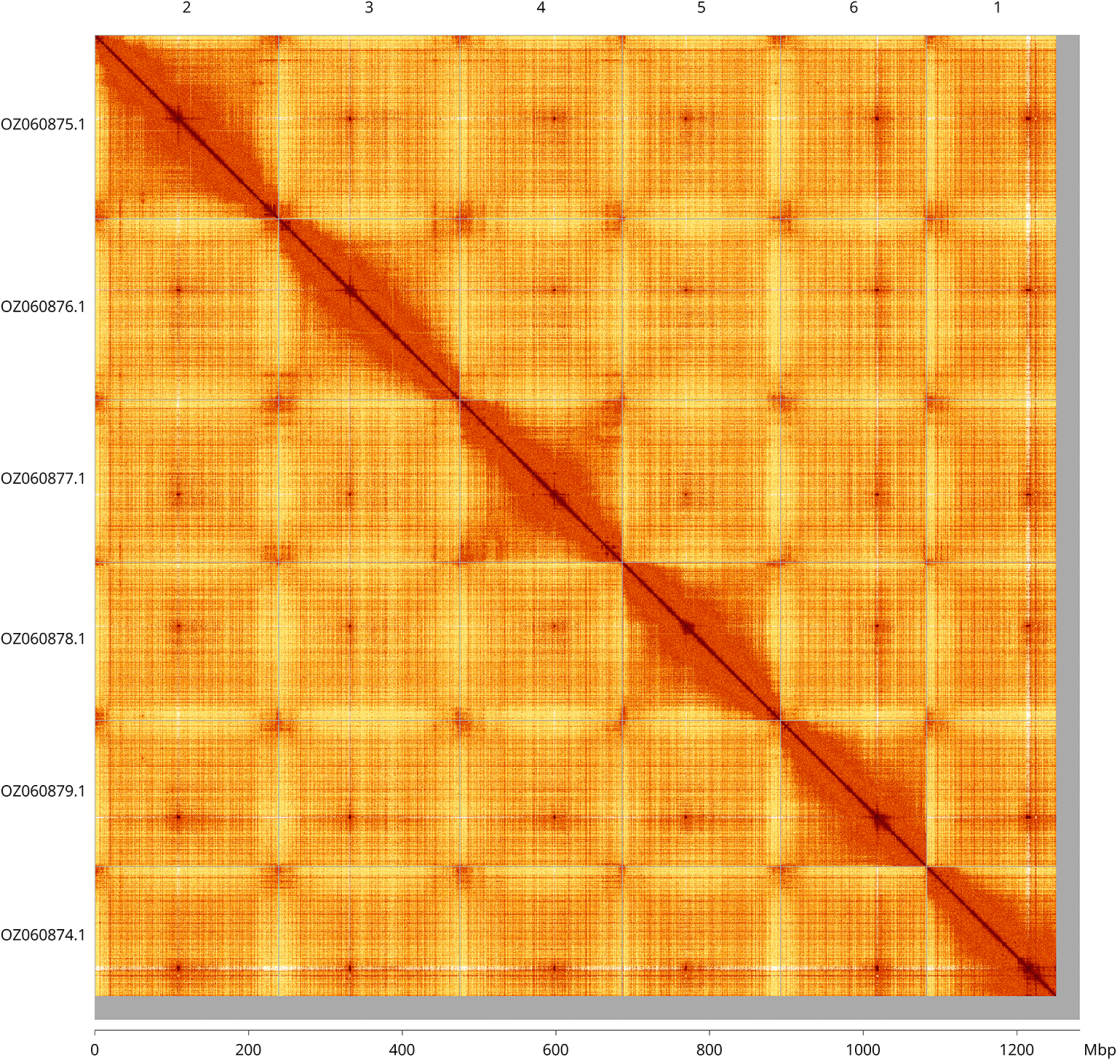
Hi-C contact map of the *Jasione montana* genome assembly. Assembled chromosomes are shown in order of size and labelled along the axes, with a megabase scale shown below. The plot was generated using PretextSnapshot.

Most of the assembly sequence (98.9%) was assigned to 6 chromosomal-level scaffolds. These chromosome-level scaffolds, confirmed by Hi-C data, are named according to size ( Figure 3; Table 3).

**Table 3.** Chromosomal pseudomolecules in the haplotype 1 genome assembly of *Jasione montana* daJasMont1.

INSDC accession	Molecule	Length (Mb)	GC%
OZ060874.1	1	185.09	36.50
OZ060875.1	2	239.47	36
OZ060876.1	3	235.78	36
OZ060877.1	4	211.23	36
OZ060878.1	5	206.26	36
OZ060879.1	6	190.06	36.50

The mitochondrial genome (length 321.12 kb, OZ060880.1) and plastid genome (length 200.38 kb, OZ060881.1) were also assembled. These sequences are included as contigs in the multifasta file of the genome submission and as standalone records.

### Assembly quality metrics

**Figure 4.**
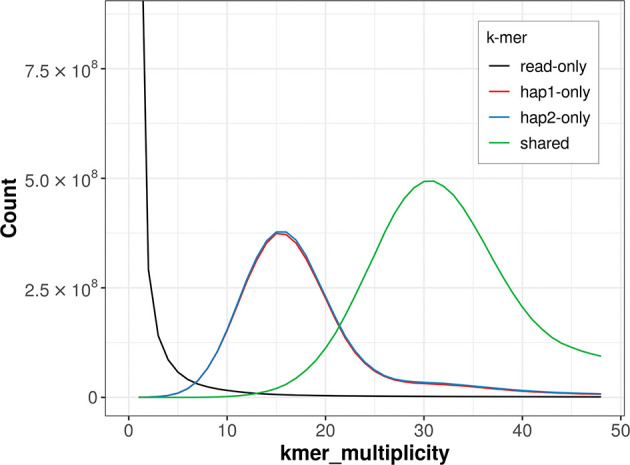
Evaluation of *k*-mer completeness using MerquryFK. This plot illustrates the recovery of *k*-mers from the original read data in the final assemblies. The horizontal axis represents *k*-mer multiplicity, and the vertical axis shows the number of *k*-mers. The black curve represents *k*-mers that appear in the reads but are not assembled. The green curve corresponds to *k*-mers shared by both haplotypes, and the red and blue curves show *k*-mers found only in one of the haplotypes.

For haplotype 1, the estimated QV is 60.7, and for haplotype 2, 60.7. When the two haplotypes are combined, the assembly achieves an estimated QV of 60.7. The *k*-mer completeness is 66.95% for haplotype 1, 67.59% for haplotype 2, and 97.97% for the combined haplotypes ( Figure 4).

**Figure 5.**
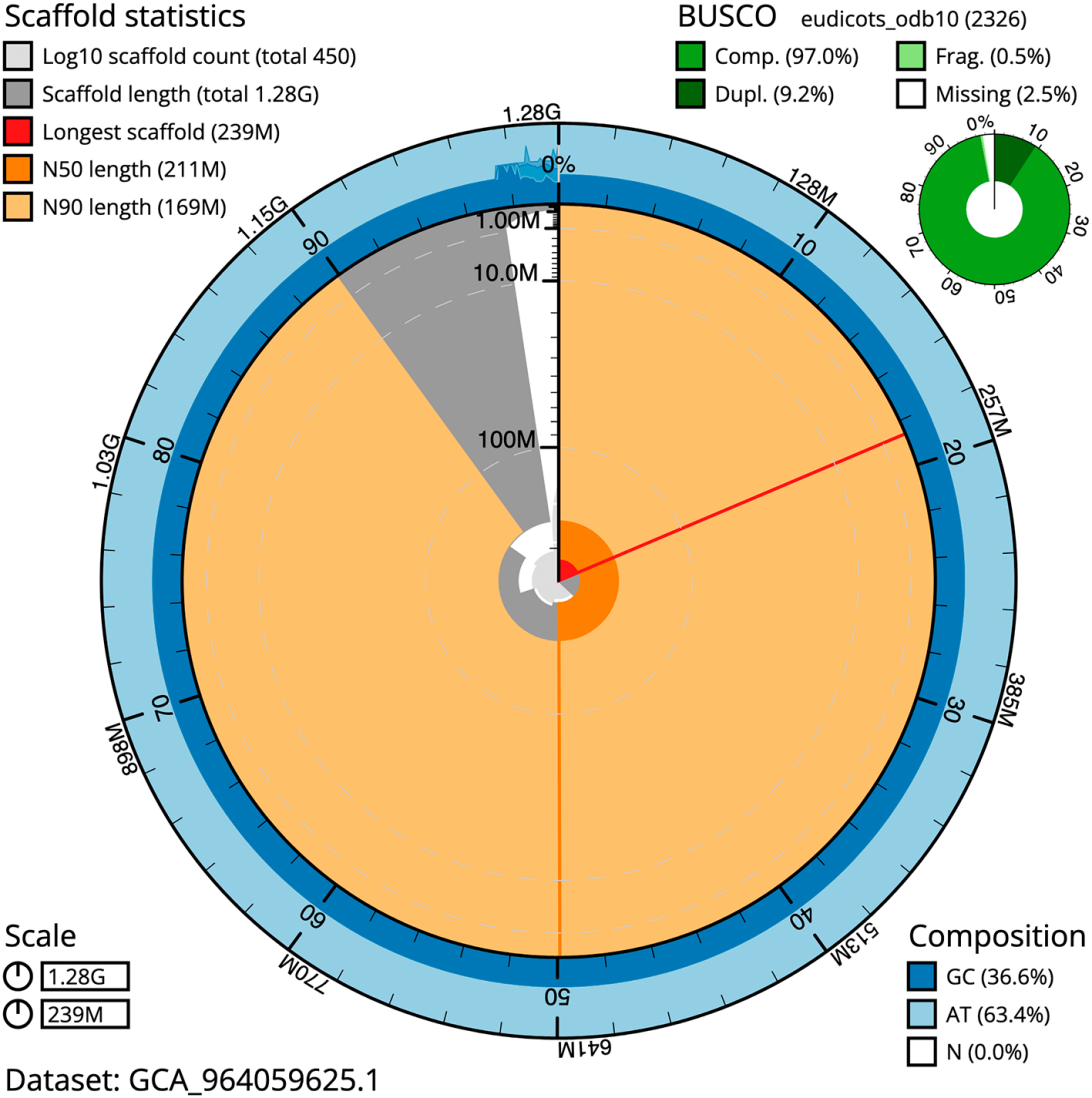
Assembly metrics for daJasMont1.hap1.1. The BlobToolKit snail plot provides an overview of assembly metrics and BUSCO gene completeness. The circumference represents the length of the whole genome sequence, and the main plot is divided into 1 000 bins around the circumference. The outermost blue tracks display the distribution of GC, AT, and N percentages across the bins. Scaffolds are arranged clockwise from longest to shortest and are depicted in dark grey. The longest scaffold is indicated by the red arc, and the deeper orange and pale orange arcs represent the N50 and N90 lengths. A light grey spiral at the centre shows the cumulative scaffold count on a logarithmic scale. A summary of complete, fragmented, duplicated, and missing BUSCO genes in the set is presented at the top right. An interactive version of this figure can be accessed on the BlobToolKit viewer.

**Figure 6.**
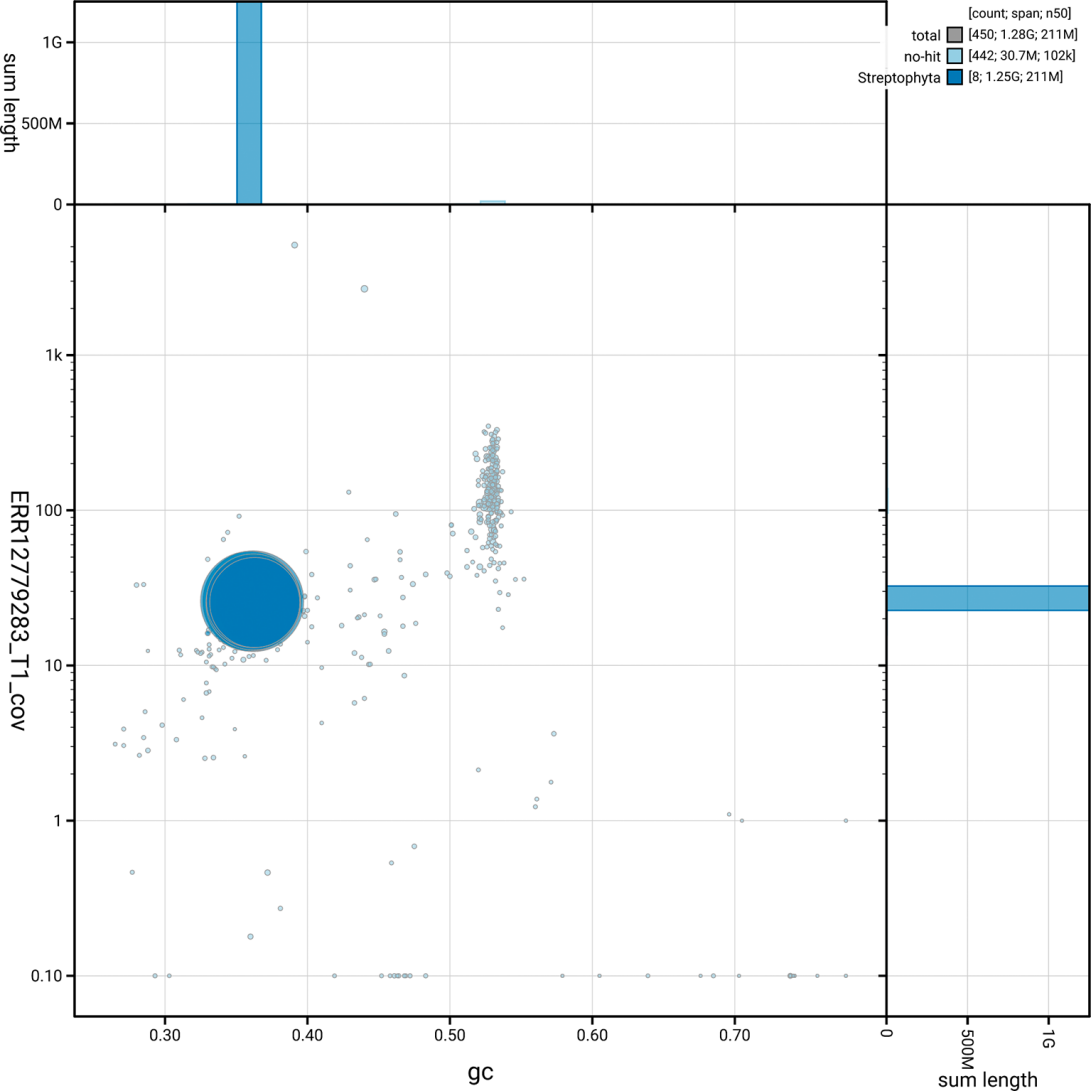
BlobToolKit blob plot for daJasMont1.hap1.1. The plot shows base coverage (vertical axis) and GC content (horizontal axis). The circles represent scaffolds, with the size proportional to scaffold length and the colour representing phylum membership. The histograms along the axes display the total length of sequences distributed across different levels of coverage and GC content. An interactive version of this figure is available on the BlobToolKit viewer.

BUSCO analysis using the eudicots_odb10 reference set ( *n* = 2 326) identified 97.0% of the expected gene set (single = 87.8%, duplicated = 9.2%) for haplotype 1. The snail plot in Figure 5 summarises the scaffold length distribution and other assembly statistics for haplotype 1. The blob plot in Figure 6 shows the distribution of scaffolds by GC proportion and coverage for haplotype 1.

Table 4 lists the assembly metric benchmarks adapted from Rhie *et al.* (2021) and the Earth BioGenome Project Report on Assembly Standards January 2026. The EBP metric calculated for the haplotype 1 is **6.C.Q60**, meeting the recommended 6.C.Q40 reference standard.

**Table 4.** Earth biogenome project summary metrics for the *Jasione montana* assembly.

Measure	Value	Benchmark
EBP summary (haplotype 1)	6.C.Q60	6.C.Q40
Contig N50 length	9.32 Mb	≥ 1 Mb
Scaffold N50 length	211.23 Mb	= chromosome N50
Consensus quality (QV)	Haplotype 1: 60.7; haplotype 2: 60.7; combined: 60.7	≥ 40
*k*-mer completeness	Haplotype 1: 66.95%; Haplotype 2: 67.59%; combined: 97.97%	≥ 95%
BUSCO	C:97.0% [S:87.8%, D:9.2%], F:0.5%, M:2.5%, n:2 326	S > 90%; D < 5%
Percentage of assembly assigned to chromosomes	98.90%	≥ 90%

**Notes:** The EBP summary uses log10(Contig N50); chromosome-level (C) or log10(Scaffold N50); Q (Merqury QV). BUSCO: C = complete; S = single-copy; D = duplicated; F = fragmented; M = missing; n = orthologues

**Table 5.** Software versions and sources used for *Jasione montana.*

Software	Version	Source
BLAST	2.14.0	ftp://ftp.ncbi.nlm.nih.gov/blast/executables/blast+/
BlobToolKit	4.3.9	https://github.com/blobtoolkit/blobtoolkit
BUSCO	5.5.0	https://gitlab.com/ezlab/busco
bwa-mem2	2.2.1	https://github.com/bwa-mem2/bwa-mem2
DIAMOND	2.1.8	https://github.com/bbuchfink/diamond
fasta_windows	0.2.4	https://github.com/tolkit/fasta_windows
FastK	1.1	https://github.com/thegenemyers/FASTK
GenomeScope2.0	2.0.1	https://github.com/tbenavi1/genomescope2.0
Gfastats	1.3.6	https://github.com/vgl-hub/gfastats
Hifiasm	0.19.8-r603	https://github.com/chhylp123/hifiasm
HiGlass	1.13.4	https://github.com/higlass/higlass
MerquryFK	1.1.0-c1	https://github.com/thegenemyers/MERQURY.FK
Minimap2	2.24-r1122	https://github.com/lh3/minimap2
Oatk	0.9	https://github.com/c-zhou/oatk
MultiQC	1.14; 1.17 and 1.18	https://github.com/MultiQC/MultiQC
Nextflow	23.04.1; 23.10.0	https://github.com/nextflow-io/nextflow
PretextSnapshot	0.0.4	https://github.com/sanger-tol/PretextSnapshot
PretextView	1.0.3	https://github.com/sanger-tol/PretextView
samtools	1.16.1; 1.17; 1.18; 1.19.2; 1.6	https://github.com/samtools/samtools
sanger-tol/ascc	0.1.0	https://github.com/sanger-tol/ascc
sanger-tol/blobtoolkit	0.6.0	https://github.com/sanger-tol/blobtoolkit
sanger-tol/curationpretext	1.4.2	https://github.com/sanger-tol/curationpretext
Seqtk	1.4-r122	https://github.com/lh3/seqtk
Singularity	3.9.0	https://github.com/sylabs/singularity
TreeVal	1.1.0	https://github.com/sanger-tol/treeval
YaHS	1.2a.2	https://github.com/c-zhou/yahs

## Author information

• Members of the Royal Botanic Gardens Kew Genome Acquisition Lab
• Members of the Plant Genome Sizing Collective
• Members of the Darwin Tree of Life Barcoding collective
• Members of the Wellcome Sanger Institute Tree of Life Management, Samples and Laboratory team
• Members of Wellcome Sanger Institute Scientific Operations – Sequencing Operations
• Members of the Wellcome Sanger Institute Tree of Life Core Informatics team
• Members of the Tree of Life Core Informatics collective
• Members of the Darwin Tree of Life Consortium

## Wellcome Sanger Institute – Legal and Governance

The materials that have contributed to this genome note have been supplied by a Darwin Tree of Life Partner. The submission of materials by a Darwin Tree of Life Partner is subject to the **‘Darwin Tree of Life Project Sampling Code of Practice’**, which can be found in full on the Darwin Tree of Life website. By agreeing with and signing up to the Sampling Code of Practice, the Darwin Tree of Life Partner agrees they will meet the legal and ethical requirements and standards set out within this document in respect of all samples acquired for, and supplied to, the Darwin Tree of Life Project. Further, the Wellcome Sanger Institute employs a process whereby due diligence is carried out proportionate to the nature of the materials themselves, and the circumstances under which they have been/are to be collected and provided for use. The purpose of this is to address and mitigate any potential legal and/or ethical implications of receipt and use of the materials as part of the research project, and to ensure that in doing so we align with best practice wherever possible. The overarching areas of consideration are:

• Ethical review of provenance and sourcing of the material
• Legality of collection, transfer and use (national and international)

Each transfer of samples is further undertaken according to a Research Collaboration Agreement or Material Transfer Agreement entered into by the Darwin Tree of Life Partner, Genome Research Limited (operating as the Wellcome Sanger Institute), and in some circumstances, other Darwin Tree of Life collaborators.

## Data Availability

European Nucleotide Archive: Jasione montana. Accession number PRJEB74169; https://identifiers.org/ena.embl/PRJEB74169. The genome sequence is released openly for reuse. The *Jasione montana* genome sequencing initiative is part of the Darwin Tree of Life Project (PRJEB40665) and the Sanger Institute Tree of Life Programme (PRJEB43745). All raw sequence data and the assembly have been deposited in INSDC databases. The genome will be annotated using available RNA-Seq data and presented through the Ensembl pipeline at the European Bioinformatics Institute. Raw data and assembly accession identifiers are reported in Tables 1 and 2. Pipelines used for genome assembly at the WSI Tree of Life are available at https://pipelines.tol.sanger.ac.uk/pipelines. Table 5 lists software versions used in this study.
