# Supplementary material for: The genome sequence of Jasione montana L., 1753 (Asterales: Campanulaceae)
Source: Wellcome Open Res. 2026 Jun 13;11:356. [Version 1] doi: 10.12688/wellcomeopenres.26778.1 (PMC13329359; doi:10.12688/wellcomeopenres.26778.1)
Supplement: Peer review history

# Reviewer report: Reviewer response for version 1

**DOI:** 10.21956/wellcomeopenres.29484.r158361

**Published:** 2026-07-02

**Authors:** Richard Michelmore, Kuntal Dey

**Affiliations:** 1 University of California Davis, Davis, California, USA

This data note provides useful documentation on the sequencing and assembly of *Jasione montana *as part of the Darwin Tree of Life Project.  As the genomes of thousands of species are assembled, it is important to have the associated metadata available as well as assemblies that are as accurate and complete as the data allow.

 The metadata provided in the introduction is good; it would be useful to have a little more taxonomic context, particularly relative to other assembled genomes in the family and species diversity of this genus.

 The materials and methods are straightforward and standard.  It is unclear whether, fresh, frozen, or dried material was used for making the PacBio and HiC libraries.  It is not stated the size of fragments that the DNA was sheared to for HiFi sequencing and the size distribution of the libraries.  The mean read length of the HiFi reads is also useful because this greatly impacts the ease of assembly.  The version of Hifiasm used should be stated.

 This genome is of high quality and exceeds the QC metrics of the Earth BioGenome project.  The amount of polishing seems to have been minimal.  Downloading the assembly and HiFi reads reveals that there are reads that span several of the gaps and therefore a little polishing could improve the assembly further.

 Importantly, there is a problem with the telomeres.  Canonical telomeric repeats are absent on one or both end of chromosome 4, 5, 6.  One of the telomeric repeats on the ends of chromosome 1 (OZ060879) has to be incorrect because there is a similar repeat on both ends, whereas these repeats should be complementary.

 They claim that it is “chromosomally complete”.  It would be more accurate to claim “chromosomally near-complete” until there is a gapless T2T assembly.  There is no mention of the NORs.  Hifiasm typically collapses these arrays into a small number of repeats representing only a fraction of the NORs.  Also, how many non-organellar contigs are not in the chromosomal scaffolds?  There are usually some; these need to be mentioned and documented.

 No attempt has been made at annotation, which would considerably increase the value of the assembly.  There are tools such as Liftoff and Helixer than can provide useful draft annotations without a lot of effort.

 In summary, this is a good data report that like the assembly could be improved with a little more polishing.

Are sufficient details of methods and materials provided to allow replication by others?

Yes

Is the rationale for creating the dataset(s) clearly described?

Yes

Are the datasets clearly presented in a useable and accessible format?

Yes

Are the protocols appropriate and is the work technically sound?

Yes

Reviewer Expertise:

Plant genomics

We confirm that we have read this submission and believe that we have an appropriate level of expertise to confirm that it is of an acceptable scientific standard, however we have significant reservations, as outlined above.

# Reviewer report: Reviewer response for version 1

**DOI:** 10.21956/wellcomeopenres.29484.r158359

**Published:** 2026-07-02

**Authors:** Panagiotis Madesis

**Affiliations:** 1 University of Thessaly, Volos, Greece

This Data Note presents a high-quality chromosome-level genome assembly of *Jasione montana* generated as part of the Darwin Tree of Life Project. The manuscript is well structured, follows the standard format adopted for Tree of Life genome reports, and provides comprehensive information regarding specimen collection, sequencing, assembly, quality assessment, and data availability. The genome assembly represents a valuable genomic resource for future comparative genomic, phylogenetic, and evolutionary studies within Campanulaceae and related taxa. 

 Some suggestions below

 The Background section could briefly expand on the broader biological significance of *Jasione montana*, particularly its ecological importance or relevance within Campanulaceae, to further highlight the value of the genomic resource beyond its inclusion in the Darwin Tree of Life initiative.

 The discrepancy between the flow cytometry genome size estimate (1,770 Mb) and the assembled genome size (1,282 Mb) is reported but only briefly discussed. Although such differences are common in highly repetitive plant genomes, one or two additional sentences commenting on possible causes (e.g. repeat-rich regions, collapsed repeats or heterozygosity) would improve interpretation for readers.

 The BUSCO analysis reports approximately 9.2% duplicated genes. While this does not diminish the quality of the assembly, a brief comment explaining whether this likely reflects biological heterozygosity, residual haplotypic duplication or lineage-specific gene duplication would provide additional context.

Are sufficient details of methods and materials provided to allow replication by others?

Yes

Is the rationale for creating the dataset(s) clearly described?

Yes

Are the datasets clearly presented in a useable and accessible format?

Yes

Are the protocols appropriate and is the work technically sound?

Yes

Reviewer Expertise:

Plant Genetics Plant Genomics, Plant molecular biology, Stress tolerance

I confirm that I have read this submission and believe that I have an appropriate level of expertise to confirm that it is of an acceptable scientific standard.

# Reviewer report: Reviewer response for version 1

**DOI:** 10.21956/wellcomeopenres.29484.r158366

**Published:** 2026-07-01

**Authors:** D Blaine Marchant

**Affiliations:** 1 University of Missouri–St. Louis, St. Louis, USA

This is a dataset article on the genome assembly of *Jasione montana *through the Darwin Tree of Life project. The article provides sufficient information on the species itself, as well as the ToL project. The tissue collection is fully transparent with photographs of the collected specimen and habitat. The methods regarding DNA extraction, sequencing, assembly, curation, and QC are thorough and the full bioinformatics pipeline is available via the Sanger ToL webiste.

 The appropriate genome statistics are provided, along with valuable tables and figures summarizing the results. I was a bit surprised that there was no RNA-sequencing for gene annotation, but it sounds like that will be provided later. One small critique is that the chromosomes in Table 3 are not named according to size as stated in the text.

 Overall, this is clearly a high-quality genome assembly that will be a valuable resource for the plant biology community.

Are sufficient details of methods and materials provided to allow replication by others?

Yes

Is the rationale for creating the dataset(s) clearly described?

Yes

Are the datasets clearly presented in a useable and accessible format?

Yes

Are the protocols appropriate and is the work technically sound?

Yes

Reviewer Expertise:

Polyploidy, plant genomics, plant reproductive development

I confirm that I have read this submission and believe that I have an appropriate level of expertise to confirm that it is of an acceptable scientific standard.
